# A practical application of a novel software solution to aid identification of research participants in primary care - the CREAM study (children with eczema antibiotic management study)

**DOI:** 10.1186/1745-6215-16-S2-P230

**Published:** 2015-11-16

**Authors:** Emma Thomas-Jones, Victoria Shepherd, Katy Addison, Kerenza Hood, Matthew Ridd, Chris Butler, Frank Sullivan, Nick Francis

**Affiliations:** 1 Cardiff University, Cardiff, UK; 2 Bristol University, Bristol, UK; 3 University of Dundee, Dundee, UK

## Background

The CREAM Study aimed to address the uncertainty around the effect of using antibiotics to treat clinically infected eczema on subjective eczema severity in children in primary care. Children were identified by GP practices and referred to the CREAM Study. A software package (Trial Torrent) that sits on primary care electronic medical record systems and can deliver pop-up reminders and templates to aid recruitment, was used within the study.

## Method

Data on problems encountered during set-up and use were recorded systematically. Participating clinicians who had TT installed were surveyed and semi-structured interviews were conducted.

## Results

Following a pilot study in 70 Scottish GP practices, TT was installed in practices participating in CREAM. Difficulties with installation, integration and operation of the software were encountered. A web-portal developed to counter these difficulties did not perform as required, therefore practices required installation of either an integrated or non-integrated (SATT) version of the software on all practice workstations potentially used by referring clinicians. Of 62 participating practices, integrated TT was installed in 26 (42%), SATT installed in 20 (32%) and 16 (26%) faxed referrals instead. Of the referrals made, only a very small proportion were sent using TT.

## Discussion

Installation of TT required commitment at the surgery for the practice manager (or delegate) to allow remote access or install the software with telephone guidance. This resulted in delays in site activation, and unnecessary / inappropriate pop ups irritated GPs, and contributed to loss of interest in the study which impacted on recruitment rates.

